# Cardiovascular Paediatric Medicines Development: Have Paediatric Investigation Plans Lost Heart?

**DOI:** 10.3390/pharmaceutics12121176

**Published:** 2020-12-02

**Authors:** Bethany Faulkner, M. Begoña Delgado-Charro

**Affiliations:** Department of Pharmacy and Pharmacology, University of Bath, Bath BA2 7AY, UK; beth.faulkner25@gmail.com

**Keywords:** paediatric investigation plan, European Medicines Agency, paediatric, valsartan, paediatric drug development, pharmaceutical, cardiovascular, heart failure, hypertension, off-label

## Abstract

This work aimed to establish whether paediatric needs in cardiovascular diseases have been met by paediatric investigation plans (PIPs) produced since the development of the European Union Paediatric Regulation in 2007. The European Medicines Agency repository was searched for patterns in the development of paediatric medicines in general. Next, positive PIPs related to cardiovascular diseases were scrutinized for outcomes and compared to specific paediatric cardiovascular needs. In total, 1866 PIPs were identified with 12% corresponding to decisions taken for cardiovascular medicines. However, despite this therapeutic area having the greatest number of overall PIPs, only 14% of established needs in paediatric cardiovascular diseases were addressed by PIPs with positive decisions. Further, 71.9% of PIPs with decisions in cardiovascular disease corresponded to full waivers, so the product would not be studied in paediatrics. Despite the progress found in overall numbers of PIPs published, cardiovascular products are still commonly used off-label in paediatrics. Particularly, there is a need to develop products to treat heart failure and hypertension, two areas with clear unmet clinical needs in paediatrics. A case study on valsartan showed that industry, regulators, health technology assessment bodies, and prescribers should work together to reduce off-label use of paediatric cardiovascular diseases (CVD).

## 1. Introduction

The development of paediatric medicines has been a long-term problem with many conditions needing treatment using medicines off-label due to lack of indications for paediatrics and appropriate formulations [1]. According to the European Medicines Agency (EMA) definition, off-label use is the “use of a medicine for an unapproved indication or in an unapproved age group, dosage or route of administration” [2]. Importantly, many paediatric patients will receive an off-label prescription during hospital stays. A survey, in the year 2000, of unlicensed and off-label use of medicines in European countries across five hospitals found that over half of paediatric patients received an off-label or unlicensed prescription [3].

In 2004, the EMA stated that the evidence demonstrating the risks of using medicines off-label in paediatrics was sufficient to trigger changes to the legislation for paediatric drug development [4]. Notably, paediatric patients receiving off-label therapies had a significantly higher risk of adverse drug reactions [5] with over a third of off-label prescriptions resulting in an adverse event. These adverse events included fatalities and hospitalization [4]. Because of the high percentage of paediatric patients receiving an off-label prescription, there is a large number of children at risk of these adverse events. Recently, an EAP/ESDPPP policy statement was published that provided an up-to-date review on the paediatric off-label medications used as well as a checklist for good practice comprising conditions that healthcare providers should consider when prescribing off-label medicines in paediatrics [6].

On 26 January 2007 the European Union introduced the European Union Paediatric Regulation (EUPR), which aimed to yield more medicines for paediatric use and better information about medicines for paediatrics and to include more paediatric patients in clinical trials [7] leading to a reduction in off-label use of medicines in this population [8]. As part of the new legislation, paediatric investigation plans (PIPs) have to be submitted with every new marketing authorisation application (MAA) to outline what the sponsor will do to investigate their product within the paediatric population. According to the EMA [7], “A PIP requirement also applies when a marketing-authorization holder wants to add a new indication, pharmaceutical form or route of administration for a medicine that is already authorized and covered by intellectual property rights”. This brings a separation between relatively new and old drugs available as generics for which investment in those three new (indication, pharmaceutical form, route of administration) scenarios is less appealing to sponsors. For “older drugs”, an incentive known as paediatric use marketing authorization (PUMA) also requiring a PIP has been made available [9].

**Figure 1:**
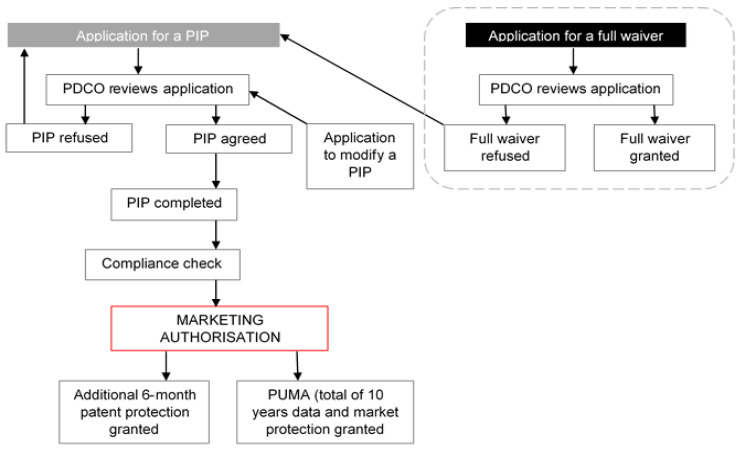
Overview of current paediatric investigation plan (PIP) application process. The flow diagram was based on the processes outlined in the standard operating procedure for PIP approvals available at European Medicines Agency (EMA) website [13] and information from the document “Enabling Development of Paediatric Medicines in Europe: 10 Years of the EU Paediatric Regulation” [14]. PUMA is a paediatric use marketing authorization granted to a product that has been developed specifically for the paediatric population.

Decisions to approve or decline PIPs are taken by the Paediatric Committee (PDCO) of the EMA and an outline for the application process can be seen in Figure 1. The PDCO can take one of six decisions (Table 1) when considering a PIP application, which are based on the pharmaceutical company’s specific request. A positive decision by the PDCO include those indicated with a P or PM abbreviation (Table 1). Pharmaceutical companies who are granted positive decisions are expected to complete new research in paediatrics for their product as agreed in the plan. Implementation of these requirements have been met with controversy by some authors and stakeholders who point at the amount of resources consumed as well as suggesting that children may be exposed to potential harm through unnecessary studies for some products [10,11,12].

In 2010, the EMA published a report [15] on a survey about off-label and unlicensed use of medicines in Europe. This report was a legal requirement of the EUPR and was based on data gathered about medicine use in paediatrics from 22 countries in Europe. This report found that anti-arrhythmic, anti-hypertensive, proton pump inhibitors, H2 antagonists, anti-asthmatic, and anti-depressant drugs were the most common classes of medicines used off-label in paediatrics. These results highlighted the therapeutic areas lacking sufficient marketing authorizations (MAs) suitable for paediatric patients and, thus, involving frequent off-label use. Importantly, common classes of drugs used to treat cardiovascular diseases were reported among those with the greatest need for paediatric development.

In order to classify paediatric medicinal needs, the EMA produced three key documents (Table 2.). The assessment of the paediatric needs (APN) list [16] was prepared by the Paediatric Working Party and comprises 21 therapeutic areas. The other two documents, the off-patent priority list (OPPL) [17] and the continuously updated inventory of paediatric medicines (IPM) [18], build upon the original APN and add new active ingredients released since its publication. In addition, it has been shown [19] that a gap exists between the therapeutic needs identified and the PIPs approved by the PDCO. In 2014, a study found that, of the 357 active substances on the APN list, only 14% had a positive PIP associated [19]. This reveals the insufficient progress in paediatric drug development that addresses previously identified therapeutic needs, despite the EUPR making PIPs mandatory for all new MA applications.

Because several classes of cardiovascular diseases (CVD) medicines have been identified in the EMA report [10] as being used commonly off-label, there is a need to investigate the current landscape of PIPs in this therapeutic area and its recent evolution. This research aimed to assess whether established paediatric CVD needs have been met by PIP applications or not.

## 2. Materials and Methods

### 2.1. Overall PIP Activity

To search for all available PIPs and filter them by decision type taken by the PDCO: “P”, “PM”, “RP”, “RPM”, “RW”, and “W” (Table 1) the EMA drug search engine [20] was used. The EMA drug search engine was preferred to other available search engines, because it contains the most up-to-date list of PIPs. In addition, it contains all the PIPs published since the legislation was enforced in 2007 and allowed the most accurate search of data needed for this research.

PIP decisions were filtered by year using 1 January to 31 December in the “date of opinion” search fields for each year. This ensured no overlap between years and enabled constructing a chronologic timeline for the data. The PIPs found were classified by decision type using the 6 decision types found in Table 1. These classifications were used to aid analysis of the data. The last PIP information recorded for this dataset was acquired on the 20 October 2019, after which this research focused in other aspects (see Section 2.2 and Section 2.3). Thus, this work provides a perspective on the overall PIP activity registered over 12 years from 1 January 2007 to 20 October 2019.

### 2.2. PIP Activity in Specific Therapeutic Areas

To search for all available PIPs and classify them by the corresponding therapeutic area, the following steps were taken: The 21 therapeutic areas used by the EMA were used to ensure that no PIPs were missed during the data search. Each therapeutic area was searched using the corresponding filter in the “therapeutic area” search criteria [20]. The PIPs found were further subdivided by decision type (Table 1) as above.

Following this, the three key documents: APN, OPPL, and IPM (Table 2) were scrutinized [16,17] The number of active substances corresponding to the same 21 therapeutic areas considered for the PIPs in each of the three documents was recorded. This enabled a direct comparison between the sets of results.

### 2.3. PIPs Addressing Paediatric Cardiovascular Needs

To assess whether established paediatric CVD needs have been met by PIP applications the following steps were taken. First, the results from the data search in the documents outlining the paediatric medicinal needs (see Section 2.2 above) were used to calculate the total number of active substances in “cardiovascular diseases” that required further development. At this stage, the name of each active substance was recorded in a separate dataset, and duplicates across the three documents listed in Table 2 were removed. Removing duplications enabled a better estimation of the number of active substances. By following these steps, the authors compiled the so-called “complete list of paediatric cardiovascular medicinal needs” (PCMN).

Next, using the EMA drug search engine [20] each active substance on the PCMN list compiled by the authors was searched for filtering by name. The EMA repository contains all PIPs produced, so searching for each active substance enabled identification of any PIPs for a product containing the relevant active substance in a straightforward manner.

Further data mining was conducted using the same EMA drug search engine but using the filters “Paediatric Investigation Plan”, “P”, “PM” in “decision type”, and “Cardiovascular diseases” in the “therapeutic area” search fields. Since all procedural steps leading to a PIP decision are recorded and stored in the EMA repository, this strategy enabled quick access to any positive PIP decisions taken. Each positive PIP was scrutinized in detail, and the data from the fields “indication”, “completion date”, and “compliance check” were recorded.

A cross-check was made between the active substances on the PCMN list, and the set of products to treat cardiovascular needs with positive PIPs (a P or PM decision) recorded. This comparison focused on active substances and products that had indications for paediatric heart failure and/or hypertension. These results from this cross-check were used to establish whether the active substances requiring paediatric development had been targeted with PIP applications. For clarity, the PIPs regarding hypertension included both systemic hypertension and pulmonary arterial hypertension, and those PIPs corresponding to heart failure did not mention a specific type of heart failure (see Supplementary Material S1-6 and S1-7).

To search for paediatric information in the summary of product characteristics (SmPCs) of CVD products with a positive PIP the following was done: The products identified previously with a positive PIP (a P or PM decision) in cardiovascular diseases (CVD) were searched by product name in the EMA repository. Where available, the summary of product characteristics (SmPC) published by the EMA for the product was scrutinized for information pertinent to paediatric patients. In particular, the information found in sections 4.2, 4.3, 4.4, 4.5, 4.8, 5.1, 5.2, and 5.3 of the product information was recorded in detail, as these are the sections where paediatric information is most commonly provided. For products without a label accessible through this repository because of the product was authorized before the EMA was established—or was not yet approved by EMA or had been approved through the national procedures—the Electronic Medicines Compendium (eMC) was searched [21]. The search was done using the product and manufacturers name, and the summary of product characteristics (SmPC) was scrutinized for the same details as above.

### 2.4. Study Limitations

This work represents a snapshot of the PIP repository between 1 January 2007 and 20 October 2019, and no updates subsequent to this date were included in the analysis. To the authors’ knowledge, all relevant information from the EMA repository was gathered, but the manual data mining process precludes absolute certainty. The analysis shown is based in the data gathered, which are available in the supplementary files, but the authors chose the aspects in which this analysis focused. For transparency, we provide all the data gathered as supplementary information so other researchers can perform other analysis, test other hypothesis focusing on other aspects, and perhaps reach different conclusions to ours. Finally, all results and conclusions have been based exclusively on the publicly available information at the EMA repository, particularly that regarding PIPs. The authors acknowledge that this is but one of the many aspects involved in paediatric medicines development and that other aspects such as clinical trials activity and positioning by health technology assessment bodies and prescribers are also crucial components in this complex area.

## 3. Results

### 3.1. Overall PIP Activity

**Figure 2:**
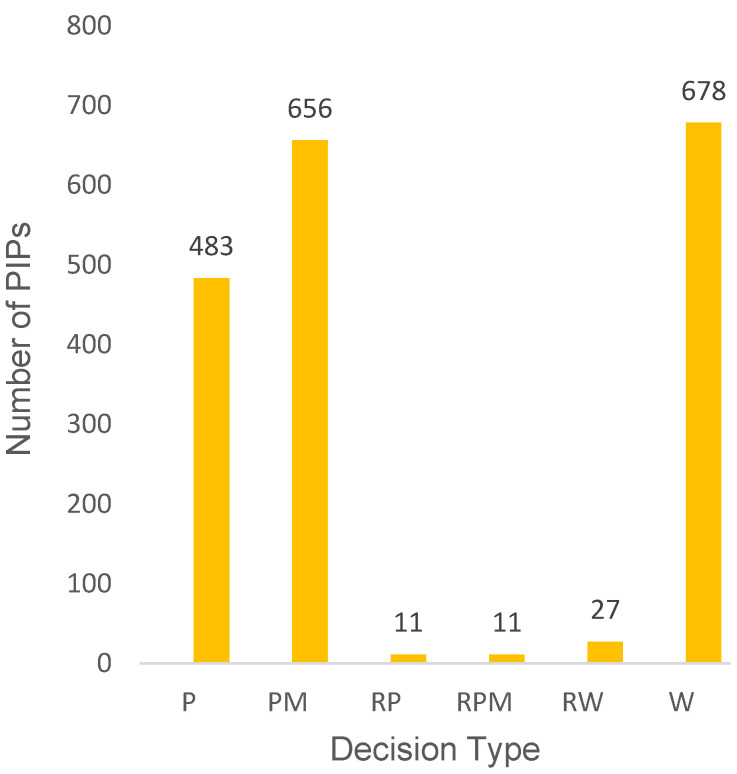
Total number of PIPs agreed by the PDCO, subdivided by decision type (see Table 1), since the EUPR was initiated and until 20 October 2019. Data taken from the EMA repository [20]. See methods for details on data mining. PIP: paediatric investigation plan; PDCO: Paediatric Committee; EUPR: European Union Paediatric Regulation; P: decision agreeing on an investigation plan with or without partial waiver(s) and or deferral(s); PM: decision on the application for modification of an agreed PIP; RP: decision refers to a refusal on a proposed paediatric investigation plan; RPM: decision refers to a refusal on the application for modification of an agreed PIP; RW: decision refers to a refusal on a request for waiver in all age groups for the listed conditions; W: decision granting a waiver in all age groups for all conditions/indications.

As expected, searching the EMA drug search engine for all available PIP decisions enabled successful identification of the total number of PIPs and of patterns in type of decision. The results showed that between January 2007, when the EUPR came into force, and 20 October 2019, the PDCO had made decisions about 1866 PIPs (see Supplementary Material, S1). Among them, the total number of positive decisions (P and PM) made up 1139 (61%) compared to waivers, which accounted for 36% of PIP decisions (Figure 2). A large number of PM decisions corresponded to products having modifications of an agreed PIP.

**Figure 3:**
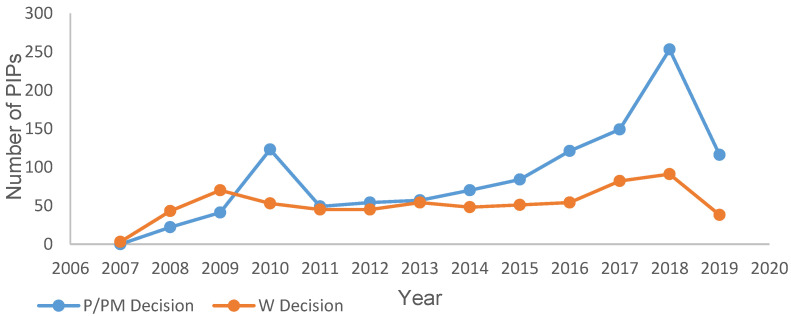
Number of positive (P/PM) PIP decisions and waivers (W) granted per year by the PDCO, since the EUPR was established in 2007. Data from 2007 and 2019 correspond to incomplete years (February to December 2007 and January to October 2019). Data taken from EMA repository [20]. See methods for details on data mining. P: decision agreeing on an investigation plan with or without partial waiver(s) and or deferral(s); PM: decision on the application for modification of an agreed PIP; W: decision granting a waiver in all age groups for all conditions/indications.

The total number of PIP decisions taken per year was studied to assess yearly fluctuations in decisions since the introduction of the EUPR. The results of this search (Figure 3) identified 2018 as the year with the highest total number of decisions made (*n* = 348) and 2008 with the lowest (*n* = 66) (see Supplementary Material, S1-2). A comparison was made between numbers of waivers and positive decisions per year; the numbers of waivers per year appeared to fluctuate less than the number of positive decisions (Figure 3). The year with the lowest number of waivers granted was 2008 (excluding the incomplete years 2007 and 2019), and the highest number of waivers was granted in 2018 (Figure 3). Positive decisions fluctuated more, with 2018 seeing 253 positive decisions and 2008 seeing 22 positive decisions. Additional peaks indicating increased activity for both waivers and positive decisions were seen for 2018 (Figure 3).

### 3.2. PIP Activity in Specific Therapeutic Areas

**Figure 4:**
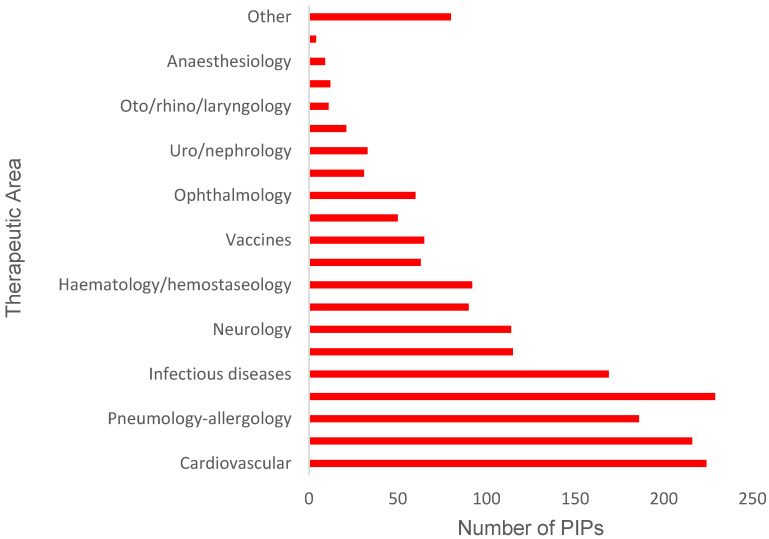
Total number of PIPs (all decision types combined) granted by the PDCO since establishment of the EUPR in 2007, subdivided by EMA therapeutic areas. Data taken from EMA repository [20]. See methods for details on data mining. PIP: paediatric investigation plan; PDCO: Paediatric Committee; EUPR: European Union Paediatric Regulation.

**Figure 5:**
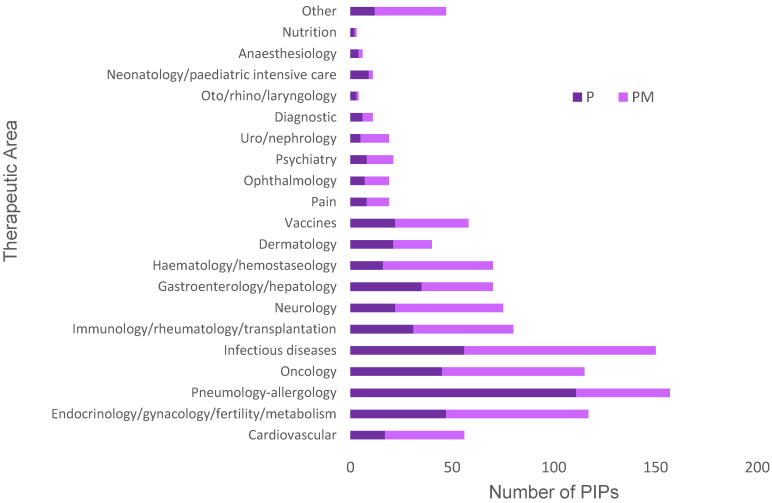
Number of positive decisions (P or PM) granted by the PDCO since the establishment of the EUPR in 2007 for the EMA therapeutic areas. Data taken from EMA repository [20]. See methods for details on data mining. PIP: paediatric investigation plan; PDCO: Paediatric Committee; EUPR: European Union Paediatric Regulation; P: decision agreeing on an investigation plan with or without partial waiver(s) and or deferral(s); PM: decision on the application for modification of an agreed PIP.

Overall PIP activity was classified into the 21 therapeutic areas used by the EMA with the results shown in Figure 4; the additional group “Other” was used for PIPs that did not belong clearly to one therapeutic area. It was found that the therapeutic areas cardiovascular diseases, endocrinology, and oncology contributed the greatest number of PIP decisions when all decision types were considered: 12.0, 11.5, and 12.2%, respectively (Figure 4). However, when PIPs in each therapeutic area were further subdivided by decision type, it was found that infectious diseases, oncology, and pneumology–allergology were the therapeutic areas with the greatest numbers of positive decisions (P or PM) (Figure 5). A detailed look into the cardiovascular diseases area revealed a relatively low number of positive decisions, as 71.9% of PIP decisions corresponded to full waivers for the product.

**Figure 6:**
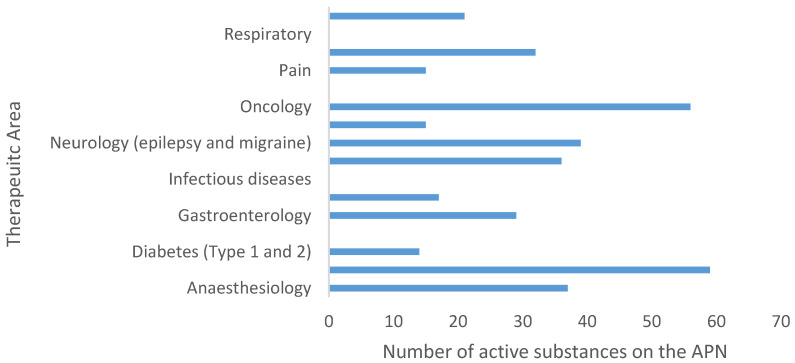
Number of active substances on the APN list per EMA therapeutic area that require additional development for paediatrics, as outlined by the Paediatric Working Party in 2007. Data taken from [16]. See methods for details on data mining.

On the other hand, analysis of the APN list indicated that most active substances requiring further development in paediatrics were in cardiovascular disease (CVD) and oncology (Figure 6). Notably, active substances to treat CVD accounted for 15% of the total number of active substances listed in the APN.

### 3.3. PIPs Addressing Paediatric Cardiovascular Needs

The total number of active substances on the PCMN, created using the APN, OPPL, and IPM documents was 61 (see Supplementary Material, S1-6). By searching those active substances by name, any positive PIPs already agreed were identified. Of the 61 active substances, nine had positive PIP decisions associated, which accounted for 14.7% of the current needs. Hypertension and heart failure were the therapeutic areas comprising the greatest number of actives (*n* = 28 and *n* = 14, respectively). Interestingly, the same therapeutic classes had the greatest number of positive PIPs associated (Table 3).

An additional EMA drug search aimed to identify innovative (i.e., products containing active substances not on the PCMN) products with positive PIPs associated was completed to identify all CVD products with a positive PIP. Assessment of the overall positive PIP activity in the therapeutic area of CVD found a total of 54 positive decisions for PIPs in this area (see Supplementary Material, S1-7). Of those PIPs, seven had compliance checks completed by the EMA, and eight PIPs had been discontinued due to lack of efficacy of the product in adults or because the MAA had not been granted/was withdrawn. A cross-check between the PCMN and all the positive PIPs addressing the indications of hypertension and heart failure revealed that 32 medicines for hypertension and heart failure (and associated conditions) had PIPs agreed (Table 4). On the whole, there was a lack of PIPs for beta blockers, calcium channel blockers, and diuretics.

Finally, the product information and labelling were scrutinized for each CVD product with a positive PIP. This was conducted to establish whether any new information had been included in the SmPC for each of the products (*n* = 54) as a result of a positive PIP. This search found that Section 4.2 (*n* = 25) and 5.1 (*n* = 23) of the SmPC contained the information most relevant to paediatric patients including both positive and negative recommendations for prescribing (Table 5).

Scrutiny of Section 4.2 of the SmPC showed an increase in the amount of clinical trial data available, as this section referred to the results presented in Section 5.1 for 16 of the products studied. Of all the product information documents scrutinized, only three provided relevant dosing information for the paediatric population (5.5%) and four recommended against prescribing the corresponding products in children. It was also found that 21 medicines with positive PIPs did not have EPARs or product information associated with them (38.9%).

## 4. Discussion

### 4.1. Overall PIP Activity

Results in Figure 2 showed a positive trend towards more decisions promoting the development of paediatric medicines, which demonstrates the success of the EUPR. The success of this legislation is further supported by other research showing that other aims of the legislation have also been achieved [14]. These include involvement of more children in clinical trials and a generation of more information about medicines to be used in paediatric patients. Whilst 36% of the total decisions found in this work were for full waivers, many of these were granted to medicines without value for the paediatric population. This is also an important aspect of the legislation, as it protects paediatric patients from participation in unnecessary clinical trials. In fact, some authors have argued this protection should be more broadly implemented [10,11,12].

In addition, Figure 3 shows a positive trend in the number of positive decisions over time. The peak time periods (see Section 3.1) demonstrate fluctuations in the overall number of approvals. Potentially, years with greater numbers of positive decisions could followed years with greater numbers of MAAs, since an MAA submission should trigger a PIP; however, this hypothesis was not tested by this work. In addition, both the overall numbers of paediatric patients involved in clinical trials and of paediatric medicines authorized have increased following introduction of the paediatric legislation in 2007 [8]. This is consistent with the increase in the number of PIPs per year since the legislation was introduced (Figure 3).

### 4.2. PIPs Approved for Specific Therapeutic Areas

Analysis of PIPs decisions for each of the 21 therapeutic areas highlighted cardiovascular diseases, endocrinology, and oncology as those with the greatest proportion of associated PIPs (Figure 4). Interestingly, these therapeutic areas are those with the most MAAs [22], which according to the paediatric legislation would prompt development of a PIP for approval or request for a waiver (Figure 1). This supports the idea that trends in paediatric drug development mirror the needs of the adult population, i.e., PIP activity follows new MAA for adults rather than MAAs addressing paediatric needs specifically. Previous research [19] has shown that, despite advances in legislation, paediatric needs are being overlooked in drug development because most new medicines are developed for adult conditions.

Compared with other therapeutic areas, cardiovascular diseases (CVD) had a large number of associated PIPs (Figure 4). It was, therefore, important to clarify whether the large PIP activity in CVD was associated with an increased number of paediatric medicines. Unfortunately, a deeper analysis of the data (Figure 5) showed that most PDCO decisions regarding CVD products involved waivers. The decision to grant a waiver means that the product will not be subject to studies in the paediatric population, as this is not expected to provide benefit to this population. It should be noted, however, that waivers are not necessarily against the interest of children; they also protect children from unnecessary participation in research, which in itself could be seen as a positive outcome. In order for a product to qualify for a full waiver, it must meet the conditions outlined by the PDCO shown in Table 6.

One potential explanation for the high numbers of full waivers granted in the CVD area is that many new CVD medicines are fixed combination therapies, i.e., two drugs administered in the same dosage form (usually a tablet) at fixed doses (see S1-8 Combination CV with waiver in the supplementary material). Whilst this approach provides benefit to the adult population, fixed combination therapies do not enable flexible dosing and are, therefore, not often suitable for the paediatric population for whom individualized dosing is important for treatment success and reduction in adverse reactions [23]. However, while the above is a reasonable hypothesis, there could be other reasons behind the observation here reported. Thus, further research is needed to elucidate why so many CVD products are granted full waivers for PIP studies. This would provide valuable information for future developments in this field, as it could give an insight into what makes a successful paediatric CVD product.

Interestingly, active substances for treating CVD had the highest requirement for development in the paediatric population (Figure 6), which identifies this therapeutic area as the one with greatest paediatric needs. Despite this, the development of paediatric CVD medicines lags behind that in other areas (Figure 5), as indicated by the fewer positive PIP decisions. The paucity of positive decisions in this area means that paediatric research has not been conducted for a significant proportion of the new CVD medicines that have reached the market recently. Further, if paediatric patients are treated with these medicines, they will be exposed to them despite the lack of paediatric safety and efficacy data generated through clinical trials. These findings indicate that future research should put greater emphasis in the CVD area to adequately meet the needs of the paediatric population and keep these vulnerable patients safe. In addition, it will be important to elucidate why this therapeutic area has lagged behind others.

In contrast, some areas (infectious diseases, oncology, and pneumology–allergology) had less overall PIPs but more positive PIPs (Figure 5). This suggests that a larger number of products in these areas offered specific therapeutic benefit for paediatric patients and, hence, the greater number of PIPs. Again, this supports the idea that patterns in drug development for paediatrics follow the trends for the adult population given the important research in these fields in recent years. In addition, previous research has shown [8] that anti-infectives represent a significant proportion of the new medicinal products centrally authorized for paediatric use following the paediatric legislation.

Given the unmet need, CVD paediatric development was considered particularly relevant, this area was analysed further and the results discussed in the next section.

### 4.3. PIPs for Paediatric Cardiovascular Needs

Results from Table 3 show that only 14.7% (nine out of 61) of identified CVD active substances with additional development needs were currently met by a positive PIP, despite this area having the greatest overall needs (Figure 6). A survey on unlicensed and off-label use of medicines [15] identified anti-hypertensives and anti-arrhythmics as two of the most common classes of medicines used off-label in Europe. This makes the lack of positive PIPs regarding these indications (Table 3 and Table 4) a particularly disappointing finding. Since anti-hypertensives and anti-arrhythmics are commonly used to treat hypertension and heart failure, this work suggests this to be an important area to direct future paediatric research.

In contrast, other medicines, which include active substances not currently identified on the PCMN, have been developed and PIP applications completed (Table 4). This suggests that the lack of paediatric development of older drugs is being slightly compensated by new drugs addressing the same indications that have arrived to the market since the original lists of paediatric needs were compiled.

Still, CVD paediatric needs have not been completely met as some of these innovative products have been discontinued, or the PIPs have been withdrawn so essential studies in children were not conducted. In addition, PDCO agreement of a PIP does not necessarily result in improved access to a specific medicine for paediatric patients. Indeed, many PIP studies have demonstrated a lack of safety and/or efficacy in the relevant populations, resulting in recommendations against prescribing the product in children (see Supplementary Material, S1-7). This is not necessarily a disappointing result, it is instead beneficial as paediatric patients’ exposure to inappropriate medicines is prevented, i.e., without these studies, a healthcare professional might have used the product off-label with a poor therapeutic outcome. Whilst this type of outcome from a PIP study does protects children, the limited access to appropriate CVD medicines still remains unsolved.

Importantly, the comparison between PIPs agreed for hypertension and heart failure (Table 4) found that there was a lack of positive or any PIPs in the critical classes of medicines (beta blockers, calcium channel blockers, and diuretics) commonly used to treat these conditions. Research has shown [1] that treatments for cardiovascular diseases represent the highest proportion of off-label prescriptions, so it is clear that the gap between paediatric needs and available authorized treatments in this therapeutic area needs to be closed. This is particularly important as the therapeutic area covers life-threatening conditions such as heart failure.

The summary of all cardiovascular drugs with an associated positive PIP (S1-7) also revealed the few number of PIPs with compliance checks. This means that the PIP studies either have not been completed or have been completed but not yet been checked for compliance by the Committee for Medicinal Products for Human Use (CHMP) of the EMA. This completion failure has an important impact on the timeline for developing paediatric medicines. Previous research [24] has shown that only 17% of medicines initially authorized for adults had completed all the required paediatric clinical trials, and that paediatric studies planned for completion following MA granting for adults were 89% less likely to be completed. This is consistent with the low number of PIPs with compliance checks found in this study and suggests that more needs to be done to ensure that sponsors complete trials and studies outlined in PIPs.

A significant proportion of drugs on the PCMN with positive PIPs did not have associated products with EPARs, meaning there was no SmPC available for examination (S1-7). This could be because the product was never submitted for regulatory assessment, or the MAA was withdrawn or not granted, for example. In addition, PIP studies could have been discontinued or the product could be awaiting a final approval decision from the EMA. Perhaps studies were completed but not published. Research has shown [24] that of all PIPs agreed by the PDCO before December 2014, 76% of studies were not completed before the initial MA in adults was granted. This highlights the need for reporting data from all clinical trials conducted in paediatrics. Data from trials are invaluable and should be reported regardless of results. If more trials are completed and more results published, steps can be made towards recommending safe doses of these medicines for children.

Results in Table 5 showed that information had been added to the SmPC for some of the CVD medicines with positive PIPs. Yet, little seems to have been done to help decisions taken by prescribers as the quality of information provided usually lacked dosing detail and specific age recommendations. The lack of recommendations for dosing of medicines in paediatrics is disappointing considering the number of additional studies completed in children. Without further recommendations for prescribing, it could be assumed that prescribers will continue to use medicines they are familiar with, as research has shown [25] that prescribers often rely on personal experience when making decisions.

A limitation of this research is that many of the PIPs had not been completed during this study time. This means that some studies were still being conducted and, therefore, posology recommendations will not be drawn until their completion. However, some PIPs had been completed and yet no recommendations were made. An area for future research would be focusing on how to conduct paediatric clinical trials that enable better data given the complexities of conducting research in this population (small numbers of participants and difficult recruitment). By doing this, improvements could be made across the therapeutic areas. Specific focus is needed on how to prove efficacy of medicines for paediatrics in clinical trials, which is a common problem for CVD clinical studies [26].

To summarize, despite the slow progress made towards development of more CVD medicines for children, there have been important advances represented by some medicines. An example of this progress is illustrated by the valsartan case study discussed below (Section 4.4).

### 4.4. Case Study: Valsartan

An example of a success for the EUPR concerns the active (brand) valsartan (Diovan). Novartis Pharma AG submitted an application on 20 August 2009 for a new pharmaceutical dosage form for their product Diovan pursuant to article 29 of the paediatric regulation [7]. This followed a positive decision on their PIP application, which included the need for a new Diovan formulation suitable for paediatrics [27]. The new MAA concerned Diovan 3 mg/1 mL, an oral solution to treat hypertension in children and adolescents (1 year to less than 18 years old). Following the successful development and testing of the product to establish its safety, efficacy, and bioequivalence, the CHMP issued a positive decision to update the MA for Diovan. In addition, the product information for Diovan 3 mg/1 mL solution includes detailed dosing instructions for the relevant age groups and information about prescribing in renal and hepatic impairment in paediatric patients [28]. It also provides details of side-effects and interactions specific to the paediatric population. Importantly, whilst Diovan 3 mg/1 mL is only licensed for paediatric patients who cannot swallow the tablets, it is the only liquid formulation of valsartan licensed in the UK. Nevertheless, it is not recommended currently by the National Institute for Health and Care Excellence (NICE) as first line treatment for hypertension in paediatric patients [29]. Whilst the British National Formulary for Children (BNFC) lists Diovan oral solution as a licensed product, reference is also made to “Forms available from special-order manufacturers include oral suspension, oral solution” [30]. This mention of specials is somehow surprising as the same formulary indicates that “Unlicensed medicines are available from ‘special-order’ manufacturers and specialist-importing companies” [31], and the MHRA recommends that “an unlicensed medicine should only be used when a patient has special requirements that cannot be met by use of a licensed medicine”. Based on the information provided by the BNFC, it was unclear to the authors which special requirements are met by specials that Diovan oral solution, licensed in the UK, cannot provide. In addition, a price comparison [32] between one month of treatment using generic valsartan tablets and the Diovan oral solution shows the new oral solution to be cheaper for a child weighing less than 35 kg (GBP 30 for tablets and GBP 9.36 for the solution). However, if the product is required as a special, further charges are added by the pharmacies dispensing the product in the UK, so the product may become more expensive for the NHS. Further clarification is needed in this area, particularly whether specials are used when licensed products are available and the reasons behind a prescribing approach, which does not benefit the patient and may become more expensive.

The positive outcome of the paediatric research conducted with valsartan resulted in a new medicine addressing the established needs for this active substance. Consequently, when the new IPM (Table 1) was written in 2013, valsartan was no longer included. This indicates the successful fulfilment of the needs on the APN (Table 3) for this product. However, this case also illustrates that paediatric patients access to medicines depends on additional, economic factors, and decisions taken by health technology assessment bodies and prescribers, which may represent the last hurdle in patients’ access to medicines. It is the opinion of the authors that medicines specifically developed for children should be given priority by Health Technology Assessment bodies (HTAs) and prescribers, even when this involves a reasonable additional cost that is justified by the improved safety and efficacy of the therapy in the paediatric population.

Further research is needed to analyse other companies’ successful development stories. This would highlight what makes a product successful and encourage other pharmaceutical companies to improve their paediatric development strategies. In addition, research is needed to discern how to conciliate patient needs with products that are cost-effective.

## 5. Conclusions

Overall, this research showed that a positive trend in the development of paediatric medicines since the establishment of the EUPR. There is, however, an imbalance between the current therapeutic needs of the paediatric population and the number of PIPs agreed across the therapeutic areas. Whilst significant advances have been made in infectious diseases, oncology, and pneumology–allergology, CVD has lagged behind. This suggests that paediatric drug development usually follows the development pipelines for adult medicines and is driven by adult medical needs; a larger focus is needed on developing medicines for specific paediatric indications.

Since the EUPR was established, some gaps in paediatric CVD needs have not been addressed. Importantly, the lack of paediatric medicines to treat hypertension and heart failure demonstrates a lack of commitment to tackle this unmet need in children. In addition, the significant number of waivers granted in CVD suggest the paucity in medicines being developed to target specific paediatric conditions.

This research aimed to identify the gaps in the therapeutic area of paediatric CVD, providing evidence needed to support future developments. As illustrated by Diovan 3 mg/1 mL, future research can bring further medicines to treat CVD in paediatrics on-label where previously treatment with off-label products was the only alternative. Tackling paediatric needs requires a concerted effort by industry and regulators, but as this case study also demonstrates, without commitment from health care authorities and prescribers, it will be challenging to reduce off-label use of paediatric CVD medicines.

## Figures and Tables

**Table 1:** Decisions that the Paediatric Committee (PDCO) can take in response to a pharmaceutical company’s request as defined by the EMA [7]. P and PM are normally referred to as “positive decisions” or “positive PIPs”.

Decision	Definition
P	Decision agreeing on an investigation plan with or without partial waiver(s) and or deferral(s)
PM	Decision on the application for modification of an agreed PIP
RP	Decision refers to a refusal on a proposed paediatric investigation plan
RPM	Decision refers to a refusal on the application for modification of an agreed PIP
RW	Decision refers to a refusal on a request for waiver in all age groups for the listed conditions
W	Decision granting a waiver in all age groups for all conditions/indications

**Table 2:** The three documents produced by the EMA outlining paediatric medicinal needs as described at the EMA website [16,17]. * Therapeutic areas in the inventory are being published one by one with 9 currently available, the first list was published in 2013, a current example of such is shown in [18].

Document	Year	Purpose
Assessment of the paediatric needs (APN)	2007	To identify active substances which require further development and/or research in paediatrics. Includes off-patent and new substances.
Off-patent priority list (OPPL)	2013	To identify priorities for development of off-patent products (INN) with the view of the developers submitting a PUMA.
Inventory of paediatric medicines (IPM)	N/A *	To detail development priorities for active substances in order to help product developers choose where to direct research

**Table 3:** Active substances (INN) for each cardiovascular indication compiled from the APN, OPPL, and IPM documents. Where an active substance appeared on more than one list it was only recorded once. The right column indicates those actives for which positive PIPs were found. Data taken from [16,17,20]. See methods for details on data mining.

Indication	Active Substances	Positive PIP Agreed
Acute hypotension	Norepinephrine	N/A
Arrhythmias	Adenosine triphosphate, amiodarone, atenolol, esmolol, flecainide, lidocaine, metoprolol, propafenone, propranolol, sotalol, verapamil	N/A
Cardiogenic shock	Arginine-vasopressin, dobutamine, dopamine, epinephrine	Dopamine (PM), dobutamine (PM)
Dyslipidaemia	Atorvastatin, colesevelam, fluvastatin, lovastatin, pravastatin, simvastatin,	N/A
Heart failure	Bisoprolol, captopril, carvedilol, dobutamine, dopamine, enalapril, enoximone, furosemide, hydrochlorothiazide/chlorothiazide, levosimendan, milrinone, nitroprusside, ramipril, spironolactone	Captopril (P), enalapril (PM), furosemide (discontinued)
Hypertension	Acebutol, amlodipine, atenolol, bosentan, candesartan, captopril, carvedilol, clonidine, dihydralazine, enalapril, esmolol, furosemide, hydrochlorothiazide/chlorothiazide, inhaled nitric oxide, irbesartan, labetalol, metoprolol, nicardipine, nifedipine, nitroprusside, prazosin, propranolol, prostacyclin, ramipril, sildenafil, spironolactone, telmisartan, valsartan	Valsartan (PM), bosentan (PM), sildenafil (PM), prostacyclin (P)
Prevention and treatment of thromboembolic events	Alteplase, aspirin, clopidogrel, dalteparin, dipyridamole, enoxaparin, heparin, low molecular weight heparin, urokinase, warfarin	Clopidogrel (PM)

**Table 4:** Active substances (INN) identified on the PCMN for heart failure and hypertension. The middle and right column identifies actives with indication in these two areas with and without agreed PIPs. Underlined actives correspond to those with agreed PIPs that appear on the APN. * Discontinued PIPs. Data taken from EMA repository [16,17,20]. See methods for details on data mining.

Indication	Agreed PIP	Not Agreed PIP
Heart failure	Captopril, dobutamine, dopamine, eleclazine *, enalapril, furosemide *, ivabradine, mavacamten, omecamtiv mecarbil, rolofylline *, sacubitril and valsartan, serelaxin *, vericiguat	Arginine-vasopressin, bisoprolol, carvedilol, enoximone, hydrochlorothiazide/chlorothiazide, levosimendan, milrinone, nitroprusside, ramipril, spironolactone
Hypertension	Aliskiren, ambrisentan azilsartan medoxomil, benzo derivative *, bosentan, clevidipine butyrate, diethanolamine, enalapril, furosemide *, imatinib, losartan potassium, macitentan, prostacyclin (and related analogues), riociguat, sildenafil, tadalafil, treprostinil, valsartan	Acebutol, amlodipine, atenolol, candesartan, captopril, carvedilol, clonidine, dihydralazine, esmolol, hydrochlorothiazide/chlorothiazide, iloprost, inhaled nitric oxide, irbesartan, labetalol, metoprolol, nicardipine, nifedipine, prazosin, propranolol, ramipril, spironolactone, telmisartan

**Table 5:** Sections of the SmPC containing relevant paediatric information for all medicines containing cardiovascular drugs with a positive PIP decision associated. Data taken from European Public Assessment Reports (EPARs) for those products [20]. See methods for details of data mining. * Products providing paediatric dosing instructions were evolucumab, rosuvastatin, and valsartan.

SmPC Section	4.2	4.3	4.4	4.5	4.8	5.1	5.2	5.2
Number of products	25 *	1	4	6	6	23	12	9

**Table 6:** Conditions for a product to be considered for a full waiver by the PDCO as stated in the EUPR [13].

A Product Can Be Granted a Full Waiver If:	
(a)	The specific medicinal product or class of medicinal products is likely to be ineffective or unsafe in part or all of the paediatric population.
(b)	The disease or condition for which the specific medicinal product or class is intended occurs only in adult populations.
(c)	The specific medicinal product does not represent a significant therapeutic benefit over existing treatments for paediatric patients.

## Supplementary Materials

The following are available online at https://www.mdpi.com/1999-4923/12/12/1176/s1, Excel file with raw data. S1 comprising Excel data sheets providing the following raw data: S1-1 Overall PIPs published by the EMA since 2007; S1-2 PIPs published per year and subdivided by decision type; S1-3 PIPs published per EMA therapeutic area and subdivided by decision type; S1-4 Number of active substances on the APN, IPL, and OPPL for each therapeutic area; S1-5 Active substances on the IPL with an indication of hypertension in paediatrics; S1-6 PCMN list compiled in this works based on the APN, IPL, and OPPL documents; S1-7 CVD medicines with associated positive PIPs and EPARs; S1-8 Combination CV with waiver.
